# Si_96_: A New Silicon Allotrope with Interesting Physical Properties

**DOI:** 10.3390/ma9040284

**Published:** 2016-04-13

**Authors:** Qingyang Fan, Changchun Chai, Qun Wei, Peikun Zhou, Junqin Zhang, Yintang Yang

**Affiliations:** 1 Key Laboratory of Ministry of Education for Wide Band-Gap Semiconductor Materials and Devices, School of Microelectronics, Xidian University, Xi’an 710071, China; ccchai@mail.xidian.edu.cn (C.C.); zhangjq@mail.xidian.edu.cn (J.Z.); ytyang@xidian.edu.cn (Y.Y.); 2 School of Physics and Optoelectronic Engineering, Xidian University, Xi’an 710071, China; qunwei@xidian.edu.cn; 3 Faculty of Science, University of Paris-Sud, Paris 91400, France; zpkhhx@gmail.com

**Keywords:** *ab initio* calculations, structural and anisotropic properties, silicon allotropes

## Abstract

The structural mechanical properties and electronic properties of a new silicon allotrope Si_96_ are investigated at ambient pressure by using a first-principles calculation method with the ultrasoft pseudopotential scheme in the framework of generalized gradient approximation. The elastic constants and phonon calculations reveal that Si_96_ is mechanically and dynamically stable at ambient pressure. The conduction band minimum and valence band maximum of Si_96_ are at the R and G point, which indicates that Si_96_ is an indirect band gap semiconductor. The anisotropic calculations show that Si_96_ exhibits a smaller anisotropy than diamond Si in terms of Young’s modulus, the percentage of elastic anisotropy for bulk modulus and shear modulus, and the universal anisotropic index *A*^U^. Interestingly, most silicon allotropes exhibit brittle behavior, in contrast to the previously proposed ductile behavior. The void framework, low density, and nanotube structure make Si_96_ quite attractive for applications such as hydrogen storage and electronic devices that work at extreme conditions, and there are potential applications in Li-battery anode materials.

## 1. Introduction

Searching for novel silicon allotropes has been of great interest over the past several decades and has been extensively studied. In other words, it is a hot topic. Experimentally, silicon has been found to have a complicated phase diagram [1]. Many semiconductor silicon structures that have an indirect band gap have been proposed, such as the *M* phase, *Z* phase [2], *Cmmm* phase [3], body-centered-tetragonal Si [4], *M*4 phase [5], lonsdaleite phase [6], *T*12 phase [7], *ST*12 phase [8], C-centered orthorhombic phase [9], *C*2/*m*-16, *C*2/*m*-20, *Amm*2, *I*-4 [10], and *P*222_1_ [11], among others. Many semiconductor silicon structures with a direct band gap have been reported, such as *P*2_1_3 phase [12], *oF*16-Si, *tP*16-Si, *mC*12-Si, and *tI*16-Si [13]. Silicon structures with metallic properties have also been reported, such as the *β*-Sn phase, *R*8 phase [14], and *Ibam* phase [15]. All of the previous studies have opened the possibility of a broader search for new allotropes of silicon that possibly exhibit novel properties. Karttunen *et al.* [16] have investigated the structural and electronic properties of various clathrate frameworks that are composed of the group 14 element semiconductors, including carbon, silicon, germanium, and tin. Several of the studied clathrate frameworks for silicon possess direct and wide band gaps [16]. Zwijnenburg *et al.* [17] have studied several new prospective low-energy silicon allotropes that use density functional theory, brute-force random search approaches and hypothetical 4CNs from Treacy and co-workers. These new low-energy silicon allotropes contain 4-member rings that were previously considered to be incompatible with low-energy silicon structures, and these computational approaches that were employed to explore the energy landscape of silicon were all found to have their own optimal zone of applicability. A systematic search for silicon allotropes was performed by employing a modified *ab initio* minima hopping crystal structure prediction method by Amsler *et al.* [18]. They found silicon clathrates that had low density and a stronger overlap of the absorption spectra with the solar spectrum compared to conventional diamond silicon, which are thus promising candidates for use in thin-film photovoltaic applications. Recently, Li *et al.* [19] found a novel cubic allotrope of carbon, C_96_-carbon, that has intriguing physical properties. We proposed Si_96_ (space group: *Pm-*3*m*), whose structure is based on C_96_-carbon [19], with silicon atoms substituting carbon atoms. The physical properties of this new cubic Si allotrope are reported in this paper. The Si_96_ is composed of six-membered silicon rings that are very similar to graphite-like six-membered carbon rings. Furthermore, the structure of the cubic Si_96_ phase is porous and has a lower density. Due to its structural porous feature and lower density, Si_96_ can also be expected to be good hydrogen storage material. The calculation of the Mulliken overlap population ensures the existence of strong covalent bonds in six-membered silicon rings, and thus, the hardness of Si_96_ is close to diamond Si. Six-membered silicon rings and zigzag six-membered silicon rings cause Si_96_ to have a lower density among the silicon allotrope materials.

## 2. Materials and Methods

All of the calculations are performed by utilizing the generalized gradient approximation (GGA) functional in the Perdew, Burke and Ernzerrof (PBE) [20] functional form in Cambridge sequential total energy package (CASTEP) [21]. The core-valence interactions were described as Ultra-soft pseudopotentials [22]. The Broyden–Fletcher–Goldfarb–Shanno (BFGS) [23] minimization scheme was used in geometric optimization. The valence electron configurations of Si 3s^2^3p^2^ are considered. A tested mesh of 4 × 4 × 4 *k*-point sampling was used for the calculations. For the new Si phase, the ultrasoft pseudopotential was used with the cutoff energy of 300 eV. The self-consistent convergence of the total energy is 5 × 10^−6^ eV/atom; the maximum force on the atom is 0.01 eV/Å, the maximum ionic displacement is within 5 × 10^−4^ Å, and the maximum stress is within 0.02 GPa. Both the HSE06 hybrid functional [24] and GGA-PBE methods were used for the calculations of electronic structures. The phonon spectra of Si_96_ used the linear response approach, also called density functional perturbation theory (DFPT), which is one of the most popular methods of an *ab initio* calculation of lattice dynamics [25].

## 3. Results and Discussion

**Figure 1:**
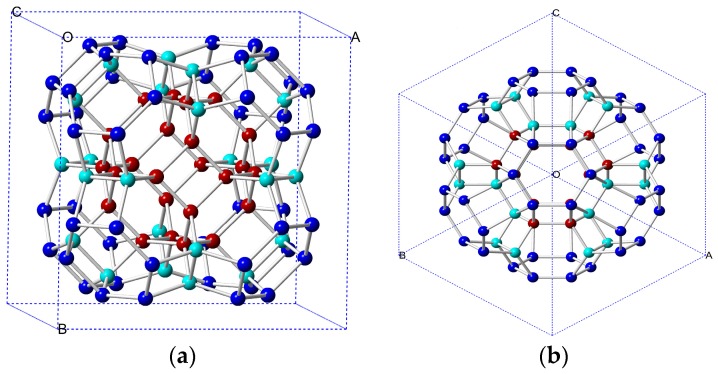
Unit cell crystal structures of Si_96_ (**a**) and along the [111] direction (**b**).

The crystal structure of Si_96_ is shown in Figure 1; it belongs to the *Pm-*3*m* space group of cubic symmetry. The basic building blocks of Si_96_ are six-membered graphite-like carbon rings, which can be clearly observed in Figure 1a. The six-membered rings are normal to the [111] direction in the structure of Si_96_ (Figure 1b). The optimized equilibrium lattice parameter is *a* = 13.710 Å at ambient pressure, and there are 96 silicon atoms in a conventional cell. The three color spheres represent three non-equivalent atoms. The blue atoms occupy the crystallographic 48*n* sites in a conventional cell, which is (0.6605, 0.7863, 0.0870). The red atoms occupy the crystallographic 24*l*_1_ sites in a conventional cell, which is (0.3792, 0.5, 0.2580). The cyan atoms also occupy the crystallographic 24*l*_2_ sites, which have the (0.5, 0.7143, 0.1373) position in a conventional cell, respectively. Furthermore, Si_96_ has nanotube-like cavities along the crystallographic main axis and the [111] direction, as shown in Figure 1a,b. It is well known that the regular arrangement of nanotubes can enhance the efficiency of the hydrogen storage of nanotubes [19]. Thus, the Si_96_ can be expected to have a good ability for hydrogen storage. Generally, the densities of the materials are closely related to the hydrogen storage ability of materials. The density (1.737 g/cm^3^) of Si_96_ is smaller than that of diamond Si (calculated value: 2.322 g/cm^3^, experimental value: 2.329 g/cm^3^). In addition, the densities of *I*-4 (2.1511 g/cm^3^), *Amm*2 (2.1809 g/cm^3^), *C*2/*m*-16 (2.2172 g/cm^3^), *C*2/*m*-20 (2.2251 g/cm^3^) [10] and P222_1_ (2.227 g/cm^3^) [11] are slightly larger than that of Si_96_. Thus, Si_96_ can be expected to have a good hydrogen storage ability among the low density materials or potential applications to Li-battery anode materials.

There are eight bond lengths in Si_96_, namely, 2.3397, 2.3398, 2.3514, 2.3557, 2.3858, 2.3873, 2.4419 and 2.4540 Å, and each bond length has a difference number; the average bond length is 2.3862 Å, which is slightly larger than that of diamond Si (2.3729 Å). The atoms occupy the crystallographic 24*l*_1_ sites, which consist of a four-membered ring. The four bond lengths are all 2.3398 Å. The other atoms occupy the crystallographic 24*l*_1_ sites, which consist of a six-membered silicon ring. The six-membered ring includes two bond lengths, 2.3514 and 2.3398 Å. Some of the atoms that occupy the crystallographic 24*l*_2_ sites are connected to occupy the crystallographic 24*l*_1_ sites and 48*n* sites, and the bond lengths are 2.3397 and 2.3873 Å, respectively. Some of the atoms that occupy the crystallographic 24*l*_2_ sites connect to themselves, and the bond length is 2.3557 Å. The other six-membered silicon ring consists of the atoms that occupy the crystallographic 48*n* sites, and it includes two bond lengths, 2.4419 and 2.4540 Å. Moreover, there is a zigzag six-membered silicon ring that consists of the three non-equivalent atoms, which includes three bond lengths, 2.3397, 2.3873 and 2.4419 Å.

**Figure 2:**
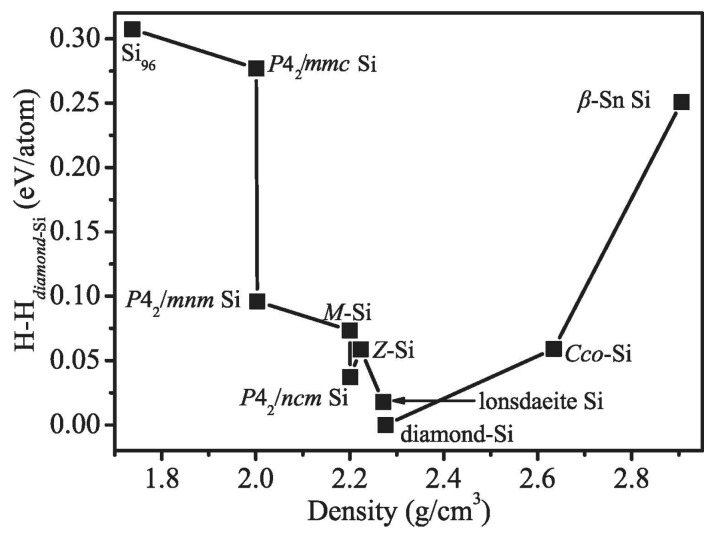
Calculated enthalpies of different silicon structures relative to the diamond Si at ambient pressure.

**Figure 3:**
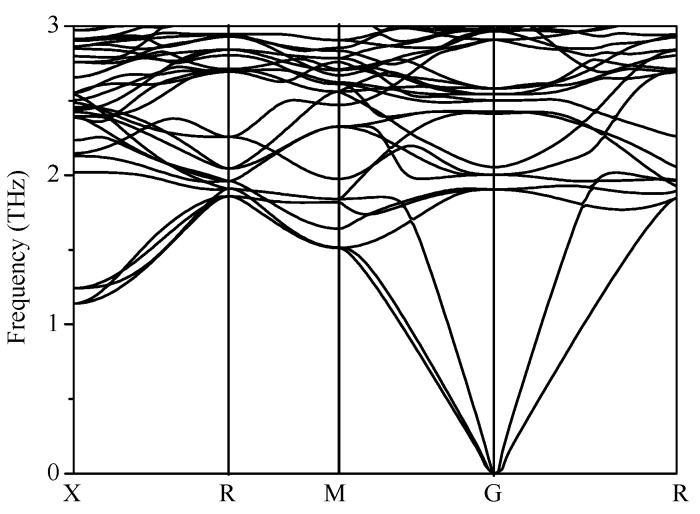
Phonon spectrum for Si_96_.

To understand the thermodynamic stability, the enthalpies of the proposed structures were compared with the experimentally known diamond Si and *β*-Sn phase Si and the theoretically proposed *M* phase, *Z* phase, lonsdaleite phase, *P*4_2_/*ncm* phase, C-centered orthorhombic (*Cco*) phase, *P*4_2_/*mnm* phase, and *P*4_2_/*mmc* phase (*tP*16-Si), as depicted in Figure 2. It is obvious that diamond Si remains the most stable phase at ambient pressure. The most unfavorable Si_96_ is higher in energy than diamond Si by 0.307 eV/atom at ambient pressure, while the metastable lonsdaleite phase is 0.017 eV/atom higher than diamond Si. In Ref. [13], the most unfavorable *tP*16-Si is higher in energy than diamond Si by 0.269 eV/atom (this work: 0.277 eV/atom) at ambient pressure. In Ref. [12], the new Si_20_ structure (space group: *P*2_1_3) is less stable than *Fd*-3*m* Si by approximately 0.3 eV/Si due to the distortion of the Si tetrahedrons. Generally, the distortion of the tetrahedron in these metastable structures leads to their higher energy. The planar four-membered silicon rings will result in a more severe distortion than five-membered silicon rings. The dynamical stability of the structure of Si_96_ was checked in this paper. The phonon dispersion for Si_96_ at ambient pressure was calculated. No imaginary frequencies are observed throughout the whole Brillouin zone, which signals dynamically the structural stability of Si_96_, as shown in Figure 3.

In addition, we calculated the hardness of Si_96_ and diamond Si using the model of Lyakhov and Oganov [26]. The hardness of Si_96_ is 9.6 GPa, which is slightly smaller than that of diamond Si (13.3 GPa). The other results of hardness for diamond Si are 8 GPa [27], 9 GPa [28], 12.4 GPa [29], and 2–16 GPa [30]. To understand the origin of the hardness, it is necessary to understand the Mulliken overlap population and bond length. The average bond length of Si_96_ is 2.3862 Å, a value that is very close to that of diamond Si, which indicates that the bonds of Si_96_ should have as high a bond strength as that of diamond. The average Mulliken overlap population of Si_96_ (0.66) is very close to that of diamond Si (0.73), which also confirms that the bond strength of Si_96_ is very strong.

We next investigate the mechanical properties of Si_96_ and diamond Si for reference. The calculated results of Si_96_ and diamond Si are listed in Table 1. The three independent elastic constants *C_ij_* of the cubic symmetry, namely *C*_11_, *C*_12_, and *C*_44_, obey the following generalized Born’s mechanical stability criteria: *C*_11_ > 0, *C*_44_ > 0, *C*_11_-*C*_12_ > 0, and *C*_11_ + 2*C*_12_ > 0 [31,32]. The elastic modulus is also calculated. Young’s modulus *E* and Poisson’s ratio *v* are taken as follows: *E* = 9*BG*/(3*B* + *G*), *v* = (3*B* − 2*G*)/[2(3*B* + *G*)]. The bulk modulus and shear modulus are smaller than that of diamond Si. The shear modulus and Young’s modulus of Si_96_ is only approximately one third that of diamond Si. In addition, the value of Poisson’s ratio is larger than that of diamond Si. Pugh [33] proposed the ratio of bulk to shear modulus (*B*/*G*) as an indication of ductile *versus* brittle characters. If *B*/*G* > 1.75, then the material behaves in a ductile way. Otherwise, the material behaves in a brittle way. The *B*/*G* of Si_96_ is larger than 1.75, which suggests that the Si_96_ allotrope is prone to ductile behavior. Interestingly, most of the silicon allotropes exhibit brittle behavior (*M*-Si: 1.22; *Z*-Si: 1.35; *T*_12_-Si: 1.56; *I*-4: 1.68; *Amm*2: 1.54, *C*2/*m*-16 Si: 1.60; *C*2/*m*-20 Si: 1.50; P222_1_ Si: 1.54 and diamond Si: 1.40), which contrasts with the previously proposed ductile behavior.

**Figure 4:**
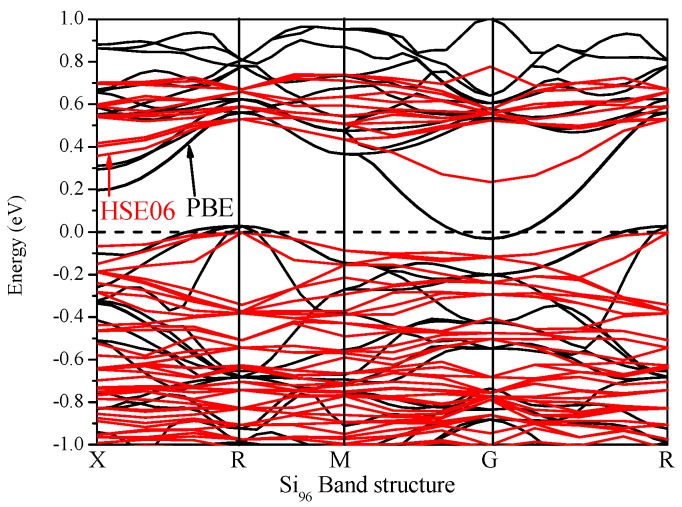
Electronic band structure using Perdew, Burke and Ernzerrof (PBE) and Heyd–Scuseria–Ernzerhof (HSE06) of Si_96_.

It is well known that the electronic structure determines the fundamental physical and chemical properties of materials. The calculated electronic band structures for Si_96_ utilizing GGA-PBE and HSE06 are presented in Figure 4. The electronic band structure calculation shows that the new silicon allotrope Si_96_ is metallic because the conduction band minimum and valence band maximum both overlap the Fermi level. It is known that the calculated band gap with DFT is usually underestimated by 30%–50%, and the true band gap must be larger than the calculated results. In consideration of this problem, Heyd *et al.* proposed a more tractable hybrid functional method, which gave rise to the Heyd–Scuseria–Ernzerhof (HSE06) functional. The hybrid functional HSE06 is used in the following form [37,38]:

(1) $$E_{xc}^{HSE}=\mu E_{x}^{HF,SR}(\omega )+(1-\mu )E_{x}^{PW91,SR}(\omega )+E_{x}^{PW91,LR}(\omega )+E_{c}^{PW91}$$

where the HF mixing parameter *μ* is 0.25, and the screening parameter that provides good accuracy for the band gaps is *ω* = 0.207 Å^−1^ [24,38]. The electronic band structure calculation shows that Si_96_ is an indirect band gap semiconductor with a band gap of 0.474 eV for the HSE06 hybrid functional. Thus, the band structure calculations show that the new Si_96_ is a narrow band gap semiconductor material.

**Figure 5:**
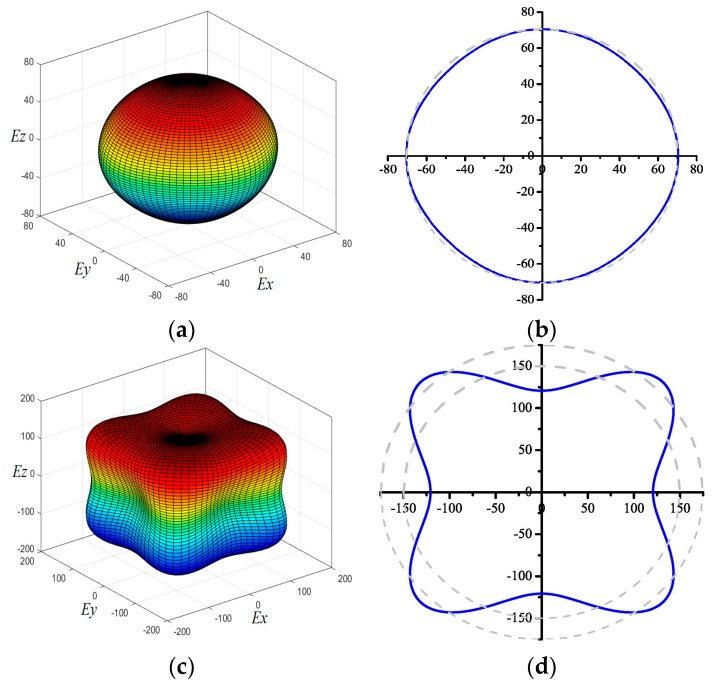
The directional dependence of Young’s modulus for Si_96_ (**a**) and diamond Si (**c**); and a 2D representation of Young’s modulus in the *xy* plane for Si_96_ (**b**) and diamond Si (**d**).

The 3D figures of the directional dependences of the reciprocals of Young’s modulus and the projections of Young’s modulus at different crystal planes for the new silicon allotrope Si_96_ and diamond Si are demonstrated in Figure 5a,c and Figure 5b,d. The three-dimensional (3D) surface construction is a valid method for describing the elastic anisotropic behavior of a solid perfectly. Usually, the anisotropic properties of materials are different due to their various crystal structures [39]. The 3D figure appears to be a spherical shape for an isotropic material, while the deviation from the spherical shape exhibits the content of anisotropy [40]. It is clear that the diamond Si exhibits greater anisotropy than that of Si_96_. The maximal and minimal value of diamond Si are 183 and 124 GPa. The maximal and minimal value of Si_96_ are 70 and 67 GPa, respectively. *E*_max_/*E*_min(diamond Si)_ is equal to 1.476 and *E*_max_/*E*_min(Si96)_ is equal to 1.048, and thus, the diamond Si exhibits greater anisotropy in Young’s modulus than that of Si_96_. In addition, the elastic anisotropy of a crystal can be depicted in many different ways. In this work, several anisotropic indices are also calculated, such as the percentage of anisotropy (*A*_B_ and *A*_G_) and the universal anisotropic index (*A*^U^). In cubic symmetry, *B*_V_ = *B*_R_ = (*C*_11_ + 2*C*_12_)/3, *G*_V_ = (*C*_11_ − *C*_12_ + 3*C*_44_)/5, and *G*_R_ = [5(*C*_11_ − *C*_12_)*C*_44_]/(4*C*_44_ + 3*C*_11_ − 3*C*_12_). The equations used can be expressed as follows: *A*_B_ = [(*B*_V_ − *B*_R_)/(*B*_V_ + *B*_R_)] × 100%, *A*_G_ = [(*G*_V_ − *G*_R_)/(*G*_V_ + *G*_R_)] × 100% and *A*^U^ = 5*G*_V_/*G*_R_ + *B*_V_/*B*_R_ − 6, and there, *A*^U^ must be greater than or equal to zero. An *A*^U^ fluctuation away from zero indicates high anisotropic elastic properties. *A*_B_ = 0, *A*_G_ = 3.58%, and *A*^U^ = 0.336 for diamond Si, and *A*_B_ = 0, *A*_G_ = 0.036%, and *A*^U^ = 0.004 for Si_96_. In other words, diamond Si exhibits greater anisotropy in Young’s modulus, *A*_B_, *A*_G_ and *A*^U^ than Si_96_.

To understand the possibility of using Si_96_ for hydrogen storage and lithium-battery anode material, we inset one and two hydrogen or lithium atoms into diamond Si (8 silicon atoms per conventional cell) and Si_96_ (96 silicon atoms per conventional cell). This method is consistent with references [5,41] to verify whether M4 silicon, diamond Si and other amorphous silicon can be used as lithium battery electrode materials. For one hydrogen (lithium) atom insertion, the volume expansion of the Si_96_ is −0.52% (−0.25%), which is much smaller than that of diamond Si (hydrogen 1.95%; lithium 2.91%, 2.94% [5]), Bct-Si (lithium 1.41% [5]) and M4-Si (lithium 1.65% [5]). For two hydrogen (lithium) atom insertions, the volume expansion of the Si_96_ is −1.18% (−0.83%), which is much smaller than that of diamond-Si (hydrogen 4.81%; lithium 7.49%, 7.53% [5]). These results show that Si_96_ has a higher capacity as a hydrogen storage and lithium-battery anode material.

## 4. Conclusions

In summary, a new silicon allotrope with space group *Pm-*3*m* is predicted, which is mechanically and dynamically stable at ambient pressure. The Si_96_ is an indirect-gap semiconductor with a band gap of 0.474 eV. The 3D surface contour of Young’s modulus is plotted to verify the elastic anisotropy of Si_96_ and diamond Si. At the same time, diamond Si exhibits greater anisotropy than Si_96_ in Young’s modulus and a slice of anisotropic indices. The void framework, low density and nanotube structures make Si_96_ quite attractive for particular applications, such as hydrogen storage and electronic devices that work at extreme conditions, and for potential applications to Li-battery anode materials.

## Figures and Tables

**Table 1:** The lattice parameters (Å), density (*ρ*: g/cm^3^), elastic constants (GPa) and elastic modulus (GPa) of Si_96_ and diamond Si.

Materials	Work	*a*	*ρ*	*C*_11_	*C*_12_	*C*_44_	*B*	*G*	*B*/*G*	*E*	*v*
Si_96_	This work	13.710	1.737	89	33	26	52	27	1.93	69	0.28
Diamond Si	This work	5.436	2.322	165	65	87	98	70	1.40	170	0.21
Diamond Si	Experimental	5.431 ^1^	2.329 ^2^	166 ^3^	64	80	102	-	-	-	-

^1^ Ref. [34] at 300 K; ^2^ Ref. [35] at 300 K; ^3^ Ref. [36].
